# What Is the Most Effective Empirical Antibiotic Treatment for Early, Delayed, and Late Fracture-Related Infections?

**DOI:** 10.3390/antibiotics11030287

**Published:** 2022-02-22

**Authors:** Susanne Baertl, Nike Walter, Ulrike Engelstaedter, Martin Ehrenschwender, Florian Hitzenbichler, Volker Alt, Markus Rupp

**Affiliations:** 1 Department for Trauma Surgery, University Hospital, 93053 Regensburg, Germany; susanne.baertl@ukr.de (S.B.); nike.walter@ukr.de (N.W.); ulengel@gmx.de (U.E.); volker.alt@ukr.de (V.A.); 2 Department for Psychosomatic Medicine, University Hospital, 93053 Regensburg, Germany; 3 Institute of Laboratory Medicine, Microbiology and Hygiene, Hospital of the Order of St. John, 93053 Regensburg, Germany; martin.ehrenschwender@barmherzige-regensburg.de; 4 Department of Infection Prevention and Infectious Diseases, University Hospital, 93053 Regensburg, Germany; florian.hitzenbichler@ukr.de

**Keywords:** fracture-related infection, antimicrobial resistance, empiric antibiotic treatment

## Abstract

Antibiotic treatment strategies for fracture-related infections (FRI) are often extrapolated from periprosthetic joint infections (PJI), although, in contrast to PJI, detailed analysis of pathogens and their antibiotic resistance is missing. Therefore, this study aimed to investigate antibiotic susceptibility profiles to identify effective empiric antibiotic treatment for early-, delayed-, and late-onset FRI. Patients treated for FRI from 2013 to 2020 were grouped into early (<2 weeks), delayed (3–10 weeks), and late (>10 weeks) onset of infection. Antibiotic susceptibility profiles were examined with respect to broadly used antibiotics and antibiotic combinations. In total, 117 patients (early *n* = 19, delayed *n* = 60, late *n* = 38) were enrolled. In early-onset FRI, 100.0% efficacy would be achieved by meropenem + vancomycin, gentamicin + vancomycin, co-amoxiclav + glycopeptide, ciprofloxacin + glycopeptide and piperacillin/tazobactam + glycopeptide. For patients with delayed FRI, the highest susceptibility was revealed for meropenem + vancomycin, gentamicin + vancomycin and ciprofloxacin + glycopeptide (96.7%). Meropenem + vancomycin was the most effective empiric antimicrobial in patients with late-onset of infection with 92.1% coverage. No subgroup differences in antibiotic sensitivity profiles were observed except for the combination ciprofloxacin + glycopeptide, which was significantly superior in early FRI (F = 3.304, *p* = 0.04). Across all subgroups meropenem + vancomycin was the most effective empiric treatment in 95.7% of patients with confirmed susceptibility. Meropenem + vancomycin, gentamicin + vancomycin, co-amoxiclav + glycopeptide are the best therapeutic options for FRI, regardless of the onset of infection. To avoid multidrug resistance, established antibiotic combinations such as co-amoxiclav with a glycopeptide seem to be reasonable as a systemic antibiotic therapy, while vancomycin + gentamicin could be implemented in local antibiotic therapy to reduce adverse events during treatment.

## 1. Introduction

In trauma surgery, reduction and internal fixation is applied to restore skeletal integrity. One of the major complications after fracture fixation utilizing metallic fracture fixation devices, is implant-related infection. Rates of developing a posttraumatic infection are reported to be around 1–2% for closed fractures ranging up to exceeding 30% for Gustilo–Anderson type III open tibia fractures [1,2]. In light of increasing numbers of fractures, especially in older adults [3], incidence of fracture–related infections (FRI) can be expected to rise as well [4,5]. The management of FRI is challenging. Depending on several factors, often multiple staged surgeries are needed for eradication of infection and finally bony consolidation [6]. Success rates only vary between 70–90% with a recurrence of the disease in 6–9% of the patients. Several limitations, such as immobility up to amputations of the affected limb, prolonged length of stay in hospital, multiple surgeries, side effects of antibiotic medication, and further socioeconomic issues, are often not to be avoided despite a variety of treatment concepts [7,8,9,10].

To classify FRI, the time of onset of symptoms after fracture fixation is commonly applied, representing time-dependent pathophysiological changes such as biofilm maturation. Thus, FRIs are differentiated as early (< 2 weeks), delayed (3–10 weeks) and late (> 10 weeks) [11]. This classification is widely adopted as it may affect treatment decisions [1,6,12,13]. For instance, implant retention may be feasible in cases of stable implants and acute infections, whereas implant exchange in a one-stage, two-stage or even multi-stage surgical treatment concept is recommended when infection is chronic, or implants are loosened. Surgical approaches are usually complemented with empiric antibiotic therapy [14], for which recommendations have been developed [15].

Recently, the microbiologic etiology in FRI has been analyzed, suggesting a similar spectrum of pathogens in early, delayed, and late FRI [16]. However, data on antimicrobial susceptibility testing and empiric antibiotic treatment strategies for FRI with respect to the onset of infection in clinical practice are still pending. In addition, treatment strategies are often extrapolated from periprosthetic joint infection (PJI) [17,18], although, in contrast to PJI, detailed analysis of pathogens and their antibiotic susceptibility/resistance is still scarce for FRI. Therefore, the purpose of this study was to answer the following question:

What is the best possible empirical antibiotic treatment for FRI cases with early-, delayed- and late-onset of infection, respectively?

## 2. Results

### 2.1. Demographics

In total, 117 patients diagnosed with FRI were included. Overall, 85 (72.6%) of the patients were male and 32 (27.4%) were female. Mean age was 55.5 ± 16.8 years. The mean BMI was 27.4 ± 5.2 kg/m^2^. Patients had comorbidities with a mean CCI of 1 (range 0–6) and a mean ASA score of 2 (range 1–4). FRI mainly occurred at the tibia (39.3%) followed by infections of the ankle (18.8%) and femur (14.5%). The mean delay from initial fracture care to onset of infection symptoms was 34.5 ± 93.5 weeks and the mean delay from symptom onset to surgical treatment for FRI was 1.3 ± 2.5 weeks. The cohort was grouped into 19 patients (16.2%) with early-onset of infection, 60 patients (51.3%) with delayed-onset of infection and 38 patients (32.5%) with late-onset of infection (Table 1). The subgroups did not differ significantly in gender (*p* = 0.8), age (*p* = 0.738), ASA score (*p* = 0.929), CCI (*p* = 0.590), BMI (*p* = 0.885) or fracture site (*p* = 0.301).

### 2.2. Empiric Antimicrobial Regimes in FRI

Methicillin-sensitive *Staphylococcus aureus* was the most frequently detected pathogen (39.7%), followed by *Staphylococcus epidermidis* (17.2%) and Gram-negative bacteria (16.4%). Difficult-to-treat microorganisms with a biofilm-active antibiotic resistance were present in 12 cases (10.3%) (Table 2). The pathogen distribution did not differ significantly between the subgroups [16].

**Figure 1:**
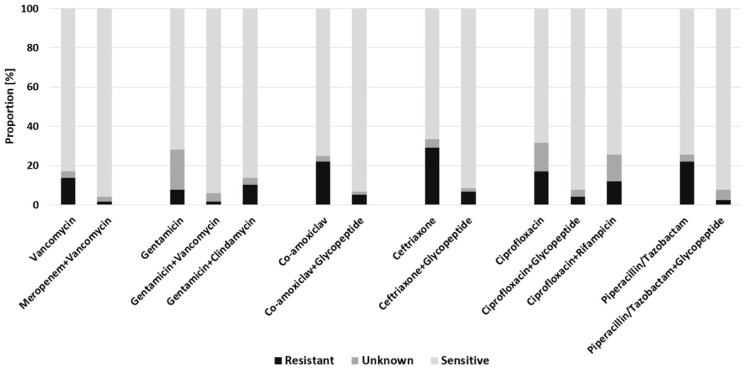
Predicted efficacy of empiric antimicrobial regimens for the whole FRI cohort.

Overall, the highest hypothetical sensitivity could be achieved by the combination of meropenem + vancomycin, with 95.7% of all patients showing confirmed susceptibility. This was followed by the combination gentamicin + vancomycin with 94.0%. More than 90% of all patients would have also been addressed by co-amoxiclav + glycopeptide (93.2%), ciprofloxacin + glycopeptide and piperacillin/tazobactam + glycopeptide (92.3% each). The lowest rates of resistance were evident for the combination gentamicin + vancomycin and meropenem + vancomycin, for which only two patients (1.7%) remained resistant due to infections with *Escherichia coli* and *Pseudomonas aeruginosa*, respectively. The highest resistance rates were found for ceftriaxone (29.1%), which could be reduced to 6.8% by an additional combination with a glycopeptide. For co-amoxiclav or piperacillin/tazobactam 22.2% of the patients would have shown resistance (Figure 1).

### 2.3. Empiric Antimicrobial Regimes in Early FRI

**Figure 2:**
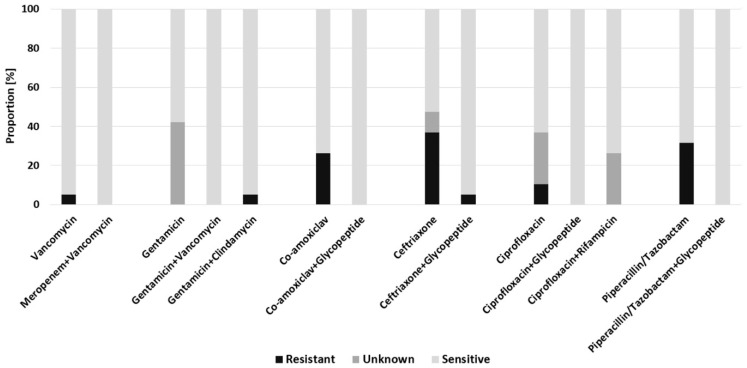
Predicted efficacy of empiric antimicrobial regimens for patients with early-onset of infection.

Comparing the predicted efficacy of empiric antimicrobial regimens between the subgroups, the combinations meropenem + vancomycin, gentamicin + vancomycin, co-amoxiclav + glycopeptide, ciprofloxacin + glycopeptide and piperacillin/tazobactam + glycopeptide would have covered all detected pathogens in early FRI and achieved 100% sensitivity in these patients (Figure 2). Monotherapies would result in resistance rates ranging from 5.0% for vancomycin up to 32.0% for piperacillin/tazobactam and 37.0% for ceftriaxone. Compared to delayed- and late-onset of infection, the only statistically significant difference was revealed regarding the combination ciprofloxacin with a glycopeptide (*F* = 3.304, *p* = 0.04), for which more patients with an early-onset of infection would have been susceptible.

### 2.4. Empiric Antimicrobial Regimes in Delayed FRI

**Figure 3:**
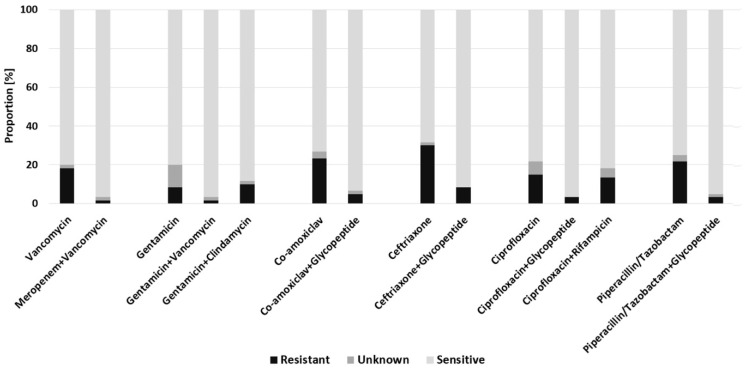
Predicted efficacy of empiric antimicrobial regimens for patients with delayed-onset of infection.

For patients with delayed-onset of infection, meropenem + vancomycin, gentamicin + vancomycin and ciprofloxacin + glycopeptide were slightly superior (96.7% coverage) than co-amoxiclav + glycopeptide (93.3%) and piperacillin/tazobactam + glycopeptide (95.0%) (Figure 3). For antibiotics typically used locally, such as vancomycin or gentamicin, sensitivity rates were 80.0% each, improved to 96.7% when both were combined. Empiric monotherapies were again inferior, while the highest resistance rates were observed for ceftriaxone (30.0%), co-amoxiclav (23.0%) and piperacillin/tazobactam (22.0%). Compared to late-onset FRI, no statistically significant difference in sensitivity rates was revealed.

### 2.5. Empiric Antimicrobial Regimes in Late-Onset FRI

**Figure 4:**
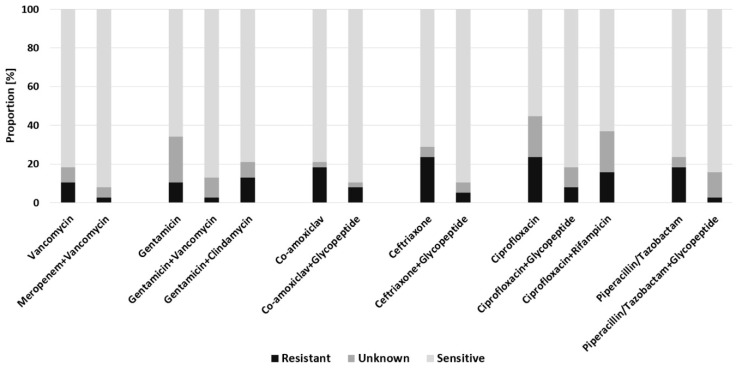
Predicted efficacy of empiric antimicrobial regimens for patients with late-onset of infection.

For patients with a late-onset of infection, highest susceptibility was found for meropenem + vancomycin (92.1%). This was followed by the combinations: ciprofloxacin + glycopeptide (89.5%); gentamicin + vancomycin (86.8%) and piperacillin/tazobactam + glycopeptide (84.2%) (Figure 4). The highest rates of resistance occurred in the empirical therapy with ceftriaxone (24.0%) or ciprofloxacin (24.0%).

## 3. Discussion

The present study compared antibiotic susceptibility testing of FRI cases with distinct onset of infection treated at a center specializing in bone and joint infection management with the purpose of evaluating best treatment options for empirical antibiotic therapy. Overall, no significant differences in the efficacy of empiric antimicrobial regimens were observed between early, delayed, and late FRI, except for early FRI, in which the combination ciprofloxacin + glycopeptide was superior compared to delayed and late FRI.

### 3.1. Empirical Antibiotic Combination Therapy Is Warranted in FRI

Current recommendations of an initial empiric broad-spectrum therapy include a lipopeptide or glycopeptide and an agent covering Gram-negative bacilli [15]. However, these guidelines targeted antibiotic treatment strategies that are currently extrapolated from PJI and even though no differences in microbiological epidemiology between PJI and FRI were reported, studies focusing on antibiotic sensitivity of pathogens in FRI are required [14,18]. Consistent with these recommendations and other reports [15,19], the combination of a glycopeptide such as vancomycin with broad-spectrum antibiotics such as meropenem achieved the highest efficacy in antimicrobial treatment of early, delayed, and late FRI. Furthermore, our results suggest that gentamicin + vancomycin, co-amoxiclav + glycopeptide, ciprofloxacin + glycopeptide and piperacillin/tazobactam + glycopeptide provide a 100% sensitivity in patients with early-onset infection, although sensitivity decreases to 90% in patients with delayed-onset infection and 80% in patients with late-onset FRI. Given the marginal hypothetical inferiority in sensitivity rates of co-amoxiclav or cephalosporin + glycopeptide, the use of broad-spectrum antimicrobial combinations such as meropenem + vancomycin or daptomycin should be limited to infections caused by multidrug-resistant Gram-negative bacteria and to patients with multiple revision procedures or septic courses of infection as part of a last-line treatment strategy [20,21]. In a previous study on orthopedic device-related infections, antimicrobial monotherapies in infection with antibiotic-resistant pathogens were leading to a significant increase in treatment failure [22]. Likewise, antibiotic monotherapies such as cephalosporins, co-amoxiclav, ciprofloxacin or piperacillin/tazobactam resulted in up to 30% resistance across all subgroups in the present cohort and therefore may not be preferable in the empiric antimicrobial therapy of FRI.

### 3.2. Downside of Antibiotic Therapy—Development of Multidrug-Resistant Pathogens

Specific data on the consumption of antibiotics in orthopedics and trauma surgery are lacking. European surveillance data suggest that 39% of infections including surgical site infections are caused by bacteria resistant to last-line antibiotics such as carbapenems [23]. In Europe as well as Germany, the burden of disease from infections with antibiotic-resistant pathogens increased significantly from 2007 to 2015. Every year, about 670,000 people in the EU suffer from infections caused by antibiotic-resistant pathogens, from which about 33,000 people die from these infections [23]. Therefore, the effective but expanding use of empirical combination therapies, including last-line antibiotics, is invariably countered by the risk of increasing occurrence of multidrug-resistant pathogens in FRI. Especially in early-onset FRI, the use of co-amoxiclav + glycopeptide or ciprofloxacin + glycopeptide seems equally effective compared to combinations containing broad-spectrum antibiotics such as piperacillin/tazobactam or meropenem. In delayed-onset FRI, our data reveal still acceptable sensitivity rates of more than 90% when co-amoxiclav or ciprofloxacin are combined with a glycopeptide. However, administration of piperacillin/tazobactam + glycopeptide resulted in relatively low sensitivity rates (84.2%) in late-onset FRI, which may indicate a possible benefit of antimicrobial combinations including meropenem in these cases. The use of daptomycin in the treatment of bone and joint infections is becoming increasingly common, but its dosage, bone penetration and ability to potentially reduce biofilm formation are currently the subject of controversial debate [20,21].

### 3.3. Side Effects of Antibiotic Therapy

A further aspect when choosing an appropriate antimicrobial agent is the consideration of possible adverse events associated with the respective drugs, especially in the setting of required long-term antimicrobial therapy. Thereby, patient-related factors such as age, concomitant diseases (e.g., chronic kidney disease) and allergies should be taken into account [24]. Valour and co-workers reported that 15% of the patients treated for bone and joint infections experienced at least one antimicrobial-related severe adverse event [25]. Vancomycin primarily entails an increased risk of nephrotoxicity, besides its more complicated management due to monitoring of serum concentrations and intravenous administration throughout therapy [25]. When combined with piperacillin/tazobactam, vancomycin was associated with a more than six-fold increase of acute renal failure in patients with PJI suggesting a synergetic toxicity of these drugs [26]. However, beta-lactam antibiotics, and particularly penicillin derivatives such as co-amoxiclav and piperacillin, were found to be most frequently involved in the occurrence of serious adverse events, including acute renal failure, hepatobiliary disorders and hematologic reactions [25,27]. Systemic administration of gentamicin carries a substantial risk of nephro- and ototoxicity, while data regarding its bone penetration indicate inconsistent results. Due to synergistic toxicity, the systemic antimicrobial therapy consisting of gentamicin + vancomycin is not recommended for bone and joint infections and should be restricted to local application [25,28,29]. In summary, clinicians should be aware of potential adverse events in the long-term treatment of bone and joint infections, particularly in the elderly and due to the risk of overdosing in obese patients [25,26]. Thus, antimicrobial susceptibility assessment is essential to allow rapid de-escalation of the initial antibiotic therapy once the pathogens and their antibiograms are identified [14]. Finally, the benefit of immediate empiric antibiotic therapy in FRI patients needs to be confirmed in further studies [30].

### 3.4. Local Antibiotic Therapy

A feasible approach to bypass unwanted side effects of systemic antibiotics, while reaching high local concentrations, is administration of local antibiotic carriers [31]. Especially, the development of new carrier materials that no longer require removal are promising in the treatment of FRI [17]. High local concentrations are particularly important since bacteria protected by biofilm formation on foreign implants and necrotic bone require substantially higher antibiotic concentrations than planktonic bacterial cells. Therefore, minimal inhibitory concentrations commonly used for antimicrobial susceptibility testing may lead to an overestimation of antibiotic efficacy at the target site, especially in chronic FRI with mature biofilm formation [32]. Traditionally used local antimicrobials include gentamicin and also vancomycin, which should be carefully considered as systemic antibiotics due to nephrotoxicity. Here, the combination of both has already been established in commercially available carrier materials (e.g., COPAL^®^ G+V), whereby an individual mixture is also possible [33,34]. Based on our results the application of gentamicin + vancomycin achieves high coverage of up to 94%, while resistance rates (1.7%) were low. In recurrent infections, higher rates of Gram-negative germs as well as polymicrobial infections should be expected [35]. Local carbapenems, which have been previously shown that they can be safely added to PMMA bone cement, could be a valuable option in these cases [36]. Hence, approaches involving vancomycin and gentamicin, but also carbapenem-carrying bone substitutes, bone cement or coated implants seem reasonable [37,38,39]. However, further studies investigating clinical outcomes, safety for the treated patient and influence of resistance profiles in microbiological environment are required.

### 3.5. Limitations

The limitations of this study are the usual suspects. First, data analysis of only one orthopedic center may lead to a local epidemiological bias. In addition, the retrospective design restricts analysis to already existing antibiograms. In some cases, antibiotic testing for certain antibiotics was sometimes not performed which leads to it being listed as “unknown” antibiotic susceptibility. This is mainly due to different panels of antibiotics available for automated and manual susceptibility testing according to the interpretative criteria released by the European Committee on Antimicrobial Susceptibility Testing (EUCAST). Further, the retrospective file analysis did not consistently allow identification of antibiotic pretreatment and its effect on the detection of infection-causing pathogens. In addition, subgroup analysis regarding the relevance of distinct anatomical localization would have been underpowered due to the low number of participants.

## 4. Materials and Methods

### 4.1. Patient Identification

A retrospective cohort study of patients treated for FRI was conducted in a level 1 trauma center in Germany. The inclusion period was defined from 1 January 2013 to 31 December 2020. Eligible patients aged 18 years or older were screened by international classification of disease (ICD) 10 diagnosis “T84.6 infection and inflammatory reaction due to internal fixation device”. Afterwards, patients’ medical charts, surgery protocols, laboratory findings as well as microbiological and histopathological reports were retrieved for inclusion criteria of FRI.

Following the 2018 international consensus meeting on musculoskeletal infection [40], FRI was confirmed by the presence of at least one of the following confirmatory criteria: (1) fistula, sinus tract or wound breakdown, (2) purulent drainage or presence of pus during surgery, (3) phenotypically indistinguishable organisms identified by culture from at least two separate deep tissue/implant specimens (including sonication fluid) and (4) histopathological findings (presence of microorganisms in deep tissue specimens or presence of >five PMN/HPF). Patients were enrolled regardless of whether they presented with primary infection or reinfection. Furthermore, patients presenting with culture-negative infections were included. If deep tissue samples or synovial fluid were not collected for microbiological analysis, patients were excluded for analysis. Patients were classified regarding the onset of infection after fracture fixation and grouped as early (0–2 weeks), delayed (3–10 weeks) and late (>10 weeks) [41].

### 4.2. Data Collection

Patient characteristics (sex, age, BMI, Charlson Comorbidity Index (CCI) [42], ASA score at the time of surgery) and details of orthopedic implant-associated infections (site of infection, type of implant and reinfection) were assessed retrospectively by reviewing electronic medical records. The microbiological database was searched for the pathogens detected and for antimicrobial susceptibility testing. Detection was either preoperatively or intraoperatively by deep tissue sampling. For polymicrobial infections, all pathogens were recorded separately.

### 4.3. Microbiology

Tissue samples were homogenized and seeded on solid and liquid culture media. All samples were incubated for 14 days. Bacteria were identified by matrix-assisted laser desorption ionization-time of flight mass spectrometry (MALDI TOF MS) using a Microflex LT mass spectrometer and BioTyper software (Bruker Daltonik, Bremen, Germany). Antibiotic susceptibility testing followed guidelines from the European Committee on Antimicrobial Susceptibility Testing (EUCAST).

### 4.4. Statistics

Descriptive and statistical data analysis was performed using the IBM SPSS Statistics software (version 24.0, IBM Corp., Armonk, NY, USA). Frequencies were expressed as numbers and percentages. Continuous parameters were presented as means ± standard deviation (SD). One-way ANOVA with Tukey- and Games–Howell post-hoc test was conducted after ensuring homogeneity of variances using Levene’s test and normal distribution by Shapiro–Wilk test. For all tests, *p*-values ≤ 0.05 were considered statistically significant.

## 5. Conclusions

In conclusion, the retrospective analysis of potential antibiotic regimens indicates that for empiric antibiotic therapy, a combination of meropenem + vancomycin, gentamicin + vancomycin, co-amoxiclav + glycopeptide, ciprofloxacin + glycopeptide or piperacillin/tazobactam + glycopeptide achieves the best hypothetical sensitivity for antimicrobial therapy in FRIs regardless of the onset of infection. Due to hitherto unknown effects on multidrug-resistance development, empirical antibiotic therapy in FRI should avoid use of reserve antibiotics such as meropenem, whenever reasonable. Established antibiotic combinations such as co-amoxiclav with a glycopeptide as systemic antibiotic therapy and vancomycin + gentamicin as local antibiotic combination should be considered as an effective antibiotic combination therapy. Meropenem instead of co-amoxiclav should be considered in patients with a septic course of infection, previous antibiotic treatment or a high risk of infection with multidrug-resistant pathogens.

## Figures and Tables

**Table 1:** Baseline characteristics of the FRI cohorts.

Characteristic	All (*n* = 117)	Early (*n* = 19)	Delayed (*n* = 60)	Late (*n* = 38)
**Demographic data**				
Sex (male)	85 (72.6%)	13 (68.4%)	43 (71.7%)	29 (76.3%)
Age (years)	55.5 ± 16.8	58.1 ± 18.7	55.4 ± 17.4	54.4 ± 15.1
BMI (kg/m^2^)	27.4 ± 5.2	28.0 ± 5.3	27.4 ± 5.2	27.2 ± 5.4
ASA score (range)	2 (1–4)	2 (1–3)	2 (1–4)	2 (1–4)
CCI (range)	1 (0–6)	1 (0–4)	1 (0–5)	1 (0–6)
**Site**				
Femur	17 (14.5%)	0	12 (20.0%)	5 (13.2%)
Shoulder	7 (6.0%)	3 (15.8%)	2 (3.3%)	2 (5.2%)
Forearm	4 (3.4%)	2 (10.5%)	2 (3.3%)	0
Hand	1 (0.9%)	0	0	1 (2.6%)
Tibia	46 (39.3%)	9 (47.4%)	20 (33.3%)	17 (44.7%)
Ankle	22 (18.8%)	3 (15.8%)	13 (21.7%)	6 (15.8%)
Foot	16 (13.7%)	1 (5.3%)	10 (16.7%)	5 (13.2%)
Spine	4 (3.4%)	1 (5.3%)	1 (1.7%)	2 (5.3%)
**Chronology of infection**				
Delay from initial fracture care to symptoms (weeks)	34.5 ± 93.5	1.3 ± 0.5	4.8 ± 2.2.	98.1 ± 145.7
Delay from symptoms to surgical treatment for FRI (weeks)	1.3 ± 2.5	1.6 ± 4.3	1.1 ± 2.0	1.5 ± 2.1
**Microbiologic documentation**				
Negative culture	11 (9.4%)	0	9 (15.0%)	2 (5.3%)
Polymicrobial infection	10 (8.6%)	3 (15.8%)	6 (10.0%)	1 (2.6%)

**Table 2:** Isolated microorganisms overall, and early, delayed and late FRI [16].

Pathogen	All (*n* = 116)	Early (*n* = 22)	Delayed (*n* = 56)	Late (*n* = 38)
*Staphylococcus aureus (MSSA)* *Staphylococcus aureus (MRSA)*	46 (39.7%) 1 (0.9%)	9 (40.9%)	22 (39.3%)	15 (39.51%) 1 (2.6%)
*Staphylococcus epidermidis*	20 (17.2%)	4 (18.2%)	9 (16.1%)	7 (18.4%)
Other *Staphylococcus* species	11 (9.5%)	3 (13.6%)	4 (7.1%)	4 (10.5%)
*Streptococcus* species	7 (6.0%)	1 (4.6%)	3 (5.4%)	3 (7.9%)
*Enterococcus* species	6 (5.2%)	2 (9.0%)	3 (5.4%)	1 (2.6%)
Gram-negative bacteria	19 (16.4%)	1 (4.6%)	13 (23.2%)	5 (13.2%)
Other	6 (5.2%)	2 (9.0%)	2 (3.6%)	2 (5.3%)

## Data Availability

The datasets analyzed during the current study are available from the corresponding author on reasonable request.
